# Enhancing Childhood Multidisciplinary Obesity Treatments: The Power of Self-Control Abilities as Intervention Facilitator

**DOI:** 10.3389/fpsyg.2018.01956

**Published:** 2018-10-16

**Authors:** Tiffany Naets, Leentje Vervoort, Sandra Verbeken, Caroline Braet

**Affiliations:** Department of Developmental, Personality and Social Psychology, Ghent University, Ghent, Belgium

**Keywords:** childhood obesity, self-control ability, obesity treatment, intervention facilitator, executive abilities/function

Currently more than 42 million children worldwide are suffering from overweight or obesity (WHO, 2016) and this number is only expected to increase to more than 70 million in 2025 (Ng et al., 2014). Both medical and psychosocial comorbidities already arise at a young age, and it also increases chances to develop adult obesity by factor five (Simmonds et al., 2016). This puts the child at risk for lifelong weight and comorbid problems (Daniels, 2009; Reilly and Kelly, 2011; Pulgaron, 2013). Moreover, childhood obesity also puts pressure on society because of its economic burden on health care (Hamilton et al., 2018). Both direct and indirect lifetime costs (e.g., caused by medical expenses and unemployment) of obesity in adolescents add up to over $254 billion in the US (Lightwood et al., 2009) and €33 billion in Europe (Fry and Finley, 2005). Recent research emphasized the importance of weight status, with more overweight leading to higher lifetime costs (Hamilton et al., 2018). Altogether we can state that childhood obesity is currently one of the most important threats worldwide (WHO, 2016; GBD Collaborators, 2017). For this reason, the World Health Organization (WHO) states that it is essential to tackle weight problems as soon as possible through evidence-based prevention and intervention programs (WHO, 2016). The recommended treatment for childhood obesity is a family-based behavioral lifestyle treatment (Oude Luttikhuis et al., 2009). The “Multidisciplinary Obesity Treatment” (MOT) combines changes in behavior, diet and physical activity with parental involvement during an intensive intervention program (Oude Luttikhuis et al., 2009). Studies of such programs show overall positive effects on BMI in children as well as in adolescents (Oude Luttikhuis et al., 2009), and that they are also cost-saving (Hollingworth et al., 2012). However, the long-term effect and possibilities for preventing adult obesity are still limited. Research suggests that weight control remains difficult, as treated children with obesity often regain weight after a longer period (Wilfley et al., 2007; Moens et al., 2010). This leaves researchers with a large responsibility to engage in longitudinal research, investigating not only long-term weight development but also how to enhance treatment effects (Mead et al., 2017).

Studying underlying mediators and moderators as intervention facilitators can be an innovative pathway to enhance treatment (Prins et al., 2015), since it allows to study if treatment programs are addressing the appropriate processes in the causal framework of the problem (Fixsen et al., 2005). Adopting a healthy lifestyle (including daily behavioral changes in eating and energy expenditure) is crucial for reaching weight control (Braet et al., 1997), but this merely implies self-control skills. Self-control, often used as a synonym for “self-regulation” in developmental literature, is a global term referring to modulating behavior, internal states, and physiological processes (Nigg, 2017). The Dual Process model describes how at least two different but intertwined cognitive systems play a role in self-control (Nigg, 2017). First, several top-down neuropsychological control processes, often referred to as “Executive Functions” (EF), activate and regulate goal-directed behavior and cognition (Riggs et al., 2012a; Diamond, 2013). “Inhibition,” “working memory,” and “cognitive flexibility” are the three main EF, referring to the ability to down-regulate dominant responses, to reactivate working memory and to flexibly switch between tasks or mental sets (Miyake et al., 2000). These top-down functions interact with the automatic bottom-up system that reacts toward the environment, of which “attention” toward certain internal states or external stimuli is the main example (Kemps et al., 2014). Literature shows four issues emphasizing the importance of self-control and its subcomponents as a promising way of facilitating weight control via obesity treatment (which will be explained below): (1) it is shown that self-control is impaired in individuals with obesity, explaining both their maladaptive eating behaviors and their weight gain, (2) these self-control deficits have been shown to hamper both short and long-term obesity treatment success, (3) although general self-control is already a component of MOT, its daily implementation remains an issue in treatment because the two underlying cognitive systems (inhibitory control and attentional processes) both show failures and (4) training these cognitive processes is possible and proven to be both effective and feasible.

First, it is known that compared to normal weight peers, individuals with obesity experience more self-control problems in resisting food-temptations in the present “obesogenic environment” where palatable high-caloric food is abundantly available (O'Brien et al., 2007). Self-control deficits make it harder to resist those temptations, contributing to the existence and sustainment of obesity (van den Bos and de Ridder, 2006). More specifically, these deficits are involved in maladaptive eating behavior (Hall, 2012; Allom and Mullan, 2014), and impressive longitudinal research even shows that self-control deficits can also predict weight gain in children as well as in adolescents (Francis and Susman, 2009; Seeyave et al., 2009; Graziano et al., 2010; Groppe and Elsner, 2017).

Second, studies show that individuals with poor self-control also struggle in obesity treatment. Children with obesity showing self-control problems (for example low inhibitory control) not only have more difficulties losing weight at both short and long term, but they also prematurely drop out more often (Nederkoorn et al., 2007). Although current childhood MOT already puts effort into targeting general self-control skills, for example by teaching daily planning and by breaking unhealthy eating habits, these vital skills hardly generalize outside the therapy setting for successful implementation in daily life (Weygandt et al., 2015).

Third, studies reveal that these generalization problems mainly exist because children with obesity cannot rely on the necessary underlying cognitive abilities (both top-down down and bottom-up) involved in sustainable health behaviors (Nederkoorn et al., 2006; Verbeken et al., 2009). As long as these cognitive abilities are not well-established, self-control will remain hampered. In obesity research, a lot of evidence has been found for both impaired inhibition and attention, in adults as well as in children with obesity. As mentioned above, *Inhibitory control* refers to the process of “thinking before acting” and suppressing undesired automatic or impulsive behaviors (Nigg, 2000), while *attentional bias* refers to the automatically captured attention toward certain stimuli (MacLeod et al., 1986), and both are involved in weight regulation (Appelhans, 2009). Similar to studies in adults (e.g., Castellanos et al., 2009; Allom and Mullan, 2014; Kemps et al., 2014), children with obesity show lower inhibitory control (Nederkoorn et al., 2006; Verbeken et al., 2009; Kamijo et al., 2012) and higher attentional biases toward unhealthy food (Yokum et al., 2011; Shank et al., 2015) in comparison to normal weight peers. The Dual Pathway view (Appelhans, 2009) theoretically explains why those processes are so important in obesity, suggesting that both an attention bias toward unhealthy food (strong bottom-up impulses), combined with inhibitory control problems (weak top-down processes) puts the individual at risk for weight issues. More specifically, maladaptive eating and sedentary behavior appear to be crucial mediating variables explaining the connection between self-control (both inhibition and attention processes) and long-term weight problems. Inhibitory control impairments in children with obesity are related with higher food intake (Riggs et al., 2012b), disinhibited or binge eating (Nederkoorn et al., 2006; Maayan et al., 2011) and lower physical activity (Riggs et al., 2012b). These results are comparable to research findings in adults with obesity (Allom and Mullan, 2014; Meule et al., 2014). Next, although research on attentional bias is more scarce than in adults (e.g., Kemps and Tiggemann, 2009; Nijs et al., 2010), children with maladaptive eating patterns (such as binge eating) exhibit attentional biases for food (Yokum et al., 2011; Shank et al., 2015; Schmidt et al., 2016). This demonstrates that specific self-control processes, such as impaired inhibition and biased attention, can be linked with crucial health behaviors concerned in weight loss.

Finally, recent findings show that those specific self-control processes are trainable (Verbeken et al., 2013). For children with obesity, our research group was the first to show increased self-control and sustainable weight loss by adding a 12-week computerized self-control ability training on top of MOT (Verbeken et al., 2013). These results were later replicated in a lab study by an independent research group (Boutelle et al., 2014). Both pilot studies provided proof-of-concept, thus emphasizing that training self-control abilities is a promising intervention facilitator in childhood obesity. Besides being effective, this type of training is also considered feasible (Verbeken et al., 2018a,b). Most children are motivated to participate, especially when conditions such as the inclusion of game elements or encouraging compliments are met (Verbeken et al., 2013; Boendermaker et al., 2015; Baranowski et al., 2016).

Based on these facts, several important directions can be stipulated for further research. First, given the relevance of training self-control skills and its underlying cognitive abilities in obese populations, additional efforts should be made to replicate the early pilot findings. Second, it will also be necessary to combine the aims of the different pilot studies and evaluate whether training both inhibition and attention concurrently can further enhance self-control behavior as predicted by the Dual Process model (Naets et al., 2018). Today, there is only one pilot study combining inhibition and attention training in adults with obesity (Stice et al., 2017). Its findings were promising, decreasing both unhealthy food reactivity and body fat. Third, evidence on long-term effects of MOT is still scarce and it remains unclear if long-term effects can be improved by adding self-control training components. For example, the childhood obesity self-control training of Verbeken et al. (2013) showed significant differences in self-control and weight loss in comparison to a control group, but there were no further weight changes after a 12-week follow-up measurement. It is clear that longer follow-up periods are necessary, because especially weight control is a long-term process and does not happen immediately after training: the effects of behavior change (i.e., healthier eating behavior, increased physical activity and decreased sedentary activity) need time to unfold in weight change. Fourth, there are indications that a sufficient number of training sessions is vital in order to obtain substantial training effects (Hakamata et al., 2010; Verbeken et al., 2018a). One hypothesis, based on the studies of Wilfley et al. (2017). and Klingberg et al. (2002) states that children should be offered enough training opportunities over a longer period. Finally, research should pay more attention to the home context in order to establish reliable and ecologically valid long-term training effects. Research should expand their focus on parental influences (Moens et al., 2006; Wilfley et al., 2007; Eichen et al., 2018). Parents are not only important role models for eating behaviors and physical activity (Golan et al., 2006), it is also shown that parenting and feeding practices indirectly contribute to the development of obesity because of their influence on how children are capable to use self-control in their energy intake and physical activity (Johnson and Birch, 1994; Moens et al., 2006). On this matter, it is yet unknown to which degree parental self-control is correlated with self-control and weight change processes in their children (Eichen et al., 2018). Since it is clear that incorporating parents is crucial in treatment and that even parent-only interventions can have an effect on weight status in children (Boutelle et al., 2011; Moens and Braet, 2012), this knowledge would also be useful to extend the effectivity of self-control training. Until now, parents were never actively involved in self-control interventions, except for limited monitoring and supervising purposes. Both investigating parental self-control and how to involve them in training will be crucial.

In sum, given the burden of obesity's large-scale consequences and the limited long-term success of current interventions, self-control is a crucial component to consider in facilitating MOT. Research not only provides evidence for the role of self-control deficits in restricted weight control, but there is also a substantial proof-of-concept for training its underlying cognitive abilities to improve success of today's MOT. This approach can be successful in enhancing daily self-control skills needed for a healthy lifestyle. As such, we strongly advocate that targeting cognitive abilities underlying self-control forms a promising and probably a necessary gateway for improving traditional childhood obesity treatment.

## Author contributions

The general theoretical content was originally conceptualized by CB, LV, and SV in accordance to previous research and the FWO-funded project. The draft of the manuscript is realized by TN as a first author, based on the work of the mentioned authors who also contributed and approved the final manuscript.

### Conflict of interest statement

The authors declare that the research was conducted in the absence of any commercial or financial relationships that could be construed as a potential conflict of interest.
